# Relationship between hepatic surgical margins of colorectal cancer liver metastases and prognosis: A review

**DOI:** 10.1097/MD.0000000000037038

**Published:** 2024-02-09

**Authors:** Xiang-Nan Ai, Qiang Zhang, Chang-Guo Jin, Hao Hu, Wen-Xuan Zhang, Zhen-Yu Wu, Dian-Rong Xiu

**Affiliations:** a Department of Hepatobiliary Surgery, Aerospace Center Hospital, Beijing, China; b Department of General Surgery, Peking University Third Hospital, Beijing, China.

**Keywords:** colorectal cancer, hepatic surgical margins, liver metastasis, prognosis

## Abstract

Colorectal cancer (CRC) remains a significant global health concern, as characterized by its high mortality rate ranking second among all the leading causes of death. The liver serves as the primary site of CRC metastasis, and the occurrence of liver metastasis is a significant contributor to mortality among patients diagnosed with CRC. The survival rate of patients with colorectal liver metastasis has significantly increased with the advancement of comprehensive tumor therapy. However, radical surgery remains the key factor. Since there are frequently multiple liver metastases, which are prone to recurrence after surgery, it is crucial to preserve as much liver parenchyma as possible without affecting the prognosis. The issue of surgical margins plays a crucial role in this regard. In this review, we begin by examining the occurrence of positive surgical margins in liver metastases of patients diagnosed with CRC. We aim to define positive margins in hepatic surgery, examine the relationship between margins and prognosis and establish a foundation for future research in this field.

## 1. Introduction

In terms of incidence among malignant tumors, colorectal cancer (CRC) has the third-highest prevalence worldwide. Approximately 1.85 million new cases of CRC are diagnosed each year. The estimated annual mortality rate for CRC exceeds 880,000, making it the second leading cause of cancer deaths worldwide.^[1–3]^ The liver is the primary site for CRC metastases, and liver metastases contribute significantly to mortality in patients with CRC. Approximately 50% of patients have been reported to develop liver metastases.^[4–6]^ Liver metastases are prevalent in about 20% to 25% of patients at the time of initial diagnosis of CRC and in 40% to 50% of patients after undergoing radical surgery for CRC.^[7,8]^ In recent years, due to the development of chemotherapeutic agents, targeted drugs, and combination therapy, the colorectal liver metastasis (CRLM) survival rate has significantly increased.^[9]^ However, radical surgery remains the most important method for achieving long-term survival in patients diagnosed with CRLM, with 5-year survival rates ranging from 40% to 60%.^[4,10,11]^ The hepatic surgical margins are essential for assessing the potential for achieving a cure. The relationship between the surgical margins and prognosis has been a controversial issue. In this paper, we examine the relationship between surgical margins and prognosis in CRLM.

## 2. Definition of positive margins

In the past, CRLM with a surgical margin of <1 cm was considered a contraindication to surgery. In other words, a surgical margin of 1 cm was considered as safe. Ekberg et al^[12]^ reported the prognostic effects of CRLM as early as 1986. They discovered that the extent of the tumor-free margin was the only prognostically relevant factor. The overall survival (OS) of patients with tumor-free margins of 1 cm or more was significantly greater compared to patients with margins of <1 cm. Bachellier et al^[13]^ conducted a retrospectively multicenter study and analyzed 1818 patients with CRLM from 1959 to 1991. The study revealed that a surgical margin of <1 cm was an independent risk factor for prognosis. They also emphasized that during surgery to treat CRLM, hepatic surgical margins of 1 cm or more should be ensured. Cady et al^[14]^ conducted a retrospective analysis of the clinical data from 244 patients diagnosed with CRLM who underwent hepatectomy. The study revealed that the surgical margin was the only statistically significant predictive factor for prognosis. In addition, both univariate and multivariate analyses determined that a 1 cm margin was a significant variable. Elias et al^[15]^ conducted a prospective study of 269 patients diagnosed with CRLM from 1984 to 1996 and discovered that a surgical margin of ≥ 1 cm was associated with a better prognosis. These studies were prompted by the observation that instances of bile duct, portal vein, hepatic vein invasion, and microsatellite lesions are typically found within a 1 cm radius of the hepatic tissue surrounding the metastases. Consequently, the “1 cm guideline” has been adhered to for quite some time.

Fong et al^[16]^ analyzed the data of 456 patients diagnosed with CRLM who underwent hepatectomy at a single center between July 1985 to December 1991. The 5-year OS rate was 38%, while for patients with positive margins (<0.1 cm), the 5-year OS rate was only 17%. This finding suggests that positive margins are an independent risk factor for prognosis. There was no statistically significant difference in prognosis between patients with negative margins <1 cm and those with margins >1 cm. As the result of a single-center retrospective study, Fong et al approached the 0.1 cm margin with extreme caution. They noted that it was difficult to obtain a 1 cm margin for multiple foci or foci in close proximity to large vessels. Kokudo et al carried out a prospective study on minimal margins for surgical resection.^[17]^ DNA mutations of K-ras and p53 were analyzed in 62 CRLM samples. The mutations K-ras and/or p53 were identified in 39 of these samples. In 2.0% (4/199) of the tested samples, hepatic parenchymal micrometastases were detected within 4 mm of the tumor margin. Micrometastases in the Glisson pedicle were more prevalent, occurring in 14.3% (3/21) of cases, and were confined to the tumor margin within 5 mm. The clinical data of 194 patients who underwent liver resection were also retrospectively analyzed. The analysis indicated that a minimum margin of 2 mm was not an independent risk factor for OS. Therefore, it can be concluded that a minimum margin of 2 mm is clinically acceptable. Pawlik et al analyzed the data of 557 patients diagnosed with CRLM who underwent hepatic resection, using a multicenter database.^[18]^ The 5-year OS rates for the 3 groups with margins of 1 to 4 mm, 5 to 9 mm, and ≥ 1 cm were determined to be 62.3%, 71.1%, and 63.0%, respectively. The difference between the groups in survival rates was not statistically significant. In addition, the 3 groups had similar overall recurrence rates (*P* = .05), and the surgical margin width had no impact on the survival rate, recurrence risk, or recurrence site. Patients with positive margins (<1 mm) had a 5-year survival rate that was significantly lower than patients with negative margins (17.1% vs 63.8%, *P* = .01). In a prospective study of 293 patients diagnosed with CRLM who underwent hepatectomy between January 1993 and December 2001, Hamady et al discovered that the 1-, 3-, 5-, and 10-year OS rates were 82%, 58%, 44%, and 36%, respectively.^[19]^ The median survival rate was 46 months. The study also revealed that histologic margin positivity (<1 mm) was a significant factor contributing to poor OS and disease-free survival (DFS) after surgery. In addition, it was also associated with a high incidence of postoperative recurrence and an overall poor survival rate. In contrast, the tumor-free margin widths of 1, 2, 5, and 10 mm had no significant impact on patient survival or recurrence rates. The aforementioned studies indicate that the poor survival in patients diagnosed with CRLM is associated with various biological characteristics of the tumor. These characteristics include the size and number of metastases, the metastatic interval, the presence of extrahepatic metastasis, and the characteristics of the lymph nodes in the primary tumor. Negative surgical margins are generally acceptable, and there is no significant difference in OS between margins measuring 0.1 to 1 cm and those exceeding 1 cm. The Expert Group on OncoSurgery Management of Liver Metastases recommended in 2015 that the minimum acceptable margin for excising CRLM be 1 mm.^[20]^ The R1 margin is defined as the distance between the tumor from the liver margin being <1 mm, under a microscope. Details about the evolution of the R1 margin are shown in Table 1.

**Table 1:** Evolution of hepatic R1 margins in CRLM.

Time	Type of research	Number of cases	Margin status	Conclusion
1986^[12]^	Single-center retrospective study	72	R1 < 10 mm	R1 is an independent risk factor for poor prognosis
1997^[13]^	Multi-center retrospective study	1818	R1 < 10 mm	R1 is an independent risk factor for poor prognosis
1998^[14]^	Single-center retrospective study	242	R1 < 10 mm	R1 is an independent risk factor for poor prognosis
1998^[15]^	Single-center prospective study	269	R1 < 10 mm	R0 has a better prognosis than R1
1997^[16]^	Single-center retrospective study	456	R1 < 1 mm R0 categorized as 1–10 mm, 10 mm or more	R1 has a poor prognosis; no difference in prognosis between 1–10 mm and 10 mm or more
2002^[17]^	Single-center retrospective study	194	<2 mm, 2–4 mm, 5–9 mm, more than 10 mm	2 mm can be used as the minimum clinically acceptable requirement
2005^[18]^	Multi-center retrospective study	557	R1 < 1 mm R0 categorized as 1–4 mm, 5–9 mm, 10 mm or more	R1 has a poor prognosis; No difference in prognosis within R0 group
2006^[19]^	Single-center prospective study	293	R1 < 1 mm	R1 is a poor factor for OS and DFS; 1, 2, 5, and 10-mm tumor-free width had no effect on OS and DFS

CRLM = colorectal cancer liver metastasis, DFS = disease free survival, OS = overall survival.

## 3. Are positive margins always undesirable

Patients who undergo R1 resection tend to have larger tumors, more lesions, and more complex surgeries, a fact that is frequently overlooked. These factors are poor prognostic indicators. Therefore, it is insufficient to attribute the low survival and high recurrence rate to the margin alone. Also, various energy devices such as cavitron ultrasonic surgical aspirator (CUSA), scalpels, bipolar coagulation, argon knife, and ultrasonic hemostat are used in liver resection. However, the use of these devices may potentially negate the prognostic value of R1 margins. Bodingbauer et al conducted a study that summarized the clinical data of 176 patients diagnosed with CRLM who underwent hepatectomy.^[21]^ The study found that the percentage of patients with multiple metastases was significantly higher in the margin-positive group (R1 < 1 mm) than in the R0 control group (*P* = .008). However, the pathological margin status did not show a significant correlation with OS (*P* = .373) and DFS (*P* = .343) in multifactorial analyses. Conversely, the histological grading of the tumor, lymph node metastasis of the primary tumor, and size and number of metastases were found to be associated with OS. It is worth noting that these adverse survival factors remained significant and were not influenced by the presence of an R1 margin. Based on their analysis, they found that the CUSA scalpel can add approximately 5 mm of additional margin, rendering the R1 margin ineffective in influencing OS or DFS. de Haas et al prospectively assessed the clinical data of 436 patients diagnosed with CRLM having a mean follow-up of 40 months.^[22]^ The R1 (<1 mm) group had more metastases in CRLM, larger tumors, a more widespread distribution across different lobes, and a higher proportion of initially unresectable cases. The 5-year OS was 61% for patients with R0 (≥1 mm) and 57% for patients with R1 (<1 mm) (*P* = .27). The 5-year DFS was 29% in the R0 group compared to 20% in the R1 group (*P* = .12). Intrahepatic recurrence was more common in the R1 group than in the R0 group (28% vs 17%; *P* = .004). Preoperative levels of carcinoembryonic antigen ≥ 10 ng/mL and more than 3 segments of hepatic resection (instead of R1 resection) were identified as independent risk factors for a lower OS rate. They used the same intraoperative CUSA knife for dissection of the hepatic parenchyma, as well as bipolar coagulation and an argon knife on the liver resection surface to control bleeding and prevent local recurrence. Herman et al conducted a review of the medical records of 91 patients diagnosed with CRLM who underwent hepatectomy.^[23]^ They analyzed the prognosis of patients with an R1 of 0 mm and found that there was no statistically significant difference in OS between patients with R1 (0 mm) and R0 (>0 mm). The DFS of patients with R1, however, was significantly lower than that of patients with R0. There was also no statistical difference between negative margins < 1 mm and ≥ 1 mm in OS and DFS. Along with the use of energy devices, they considered the role of perioperative chemotherapy. Montalti et al examined the correlation between R1 margins (<1 mm) and prognosis following laparoscopic surgery for CRLM.^[24]^ They discovered that recurrence-free survival rate was significantly lower in the R1 resection group (*P* = .038). However, OS did not differ significantly from the R0 group. They also suggested that the lack of a difference in overall OS could be attributed to repeated hepatic resection following recurrence. In addition, the presence of 2 or more lesions was identified as a risk factor for R1. Pencovich et al concluded that although R1 resection was associated with more severe disease behavior and postoperative complications, the long-term prognosis of patients after R1 resection was no worse than those who underwent R0 resection.^[25]^ Also, DFS did not differ significantly from R0 resection.

The implementation of preoperative neoadjuvant chemotherapy, as revealed by some studies, has suggested that the diversified treatment of tumors currently has made it possible to resect many previously unresectable tumors. In addition, postoperative adjuvant chemotherapy, the use of molecularly targeted agents, and the adjuvant function of immunotherapy have been found to counteract the negative prognostic effects of R1 resection margins. Truant et al observed 273 patients (214 R0/59 R1) admitted between 2000 and 2010 and compared R0 resection with R1 resection.^[26]^ They discovered that R1 resection was associated with lower 5-year survival (39.1% vs 54.2%, *P* = .010), DFS (15.2% vs 31.1%, *P* = .021), and progression-free survival (33.1% vs 47.3%, *P* = .033). After conducting propensity score matching, they identified several independent risk factors associated with poor survival. These factors included the number, size, and short time interval of liver metastases, lymph node status, rectal primary lesion, lack of adjuvant chemotherapy, and absence of R1 resection. These results indicate that these factors are indicative of the aggressive biological behavior of the tumor. In addition, since 2005, with the implementation of more systematic chemotherapy regimens, the disparity in DFS between the groups has decreased (*P* = .081), and the discrepancy in progression-free survival has vanished (*P* = .264). They concluded that the status of the R1 margin is not an independent predictor of survival, but rather an indicator of advanced and/or more severe disease. They also found that the use of adjuvant chemotherapy reduced the difference in R1 and R0 status between the groups. Margonis et al concluded that in the modern era of systemic chemotherapy, the impact of margin status on prognosis appears to be minimal in comparison to patient and tumor factors.^[27]^ They also discovered that re-excision of R1 margins to achieve R0 status does not enhance the long-term prognosis of the patient, even when R1 resection is present. Due to the use of systemic chemotherapy, R1 resection is not a risk factor for OS and DFS. Tanaka et al concluded that among the 310 patients studied, 55 R1-resected patients had worse DFS (*P* < .01) and OS (*P* < .01) than 255 R0-resected patients.^[28]^ However, among those tumors that were initially unresectable or barely resectable and underwent preoperative chemotherapy, there was no significant difference in DFS (*P* = .44) and OS (*P* *=* .50) between the 73 R0-resected patients and 36 R1-resected patients. Upon administration of neoadjuvant therapy, the difference between the R1 and R0 groups disappeared. Sasaki et al conducted a study on 630 patients who underwent surgery for CRLM at Johns Hopkins Hospital between 2000 and 2015.^[29]^ The patients were divided into 2 groups based on whether or not they were treated with bevacizumab. Among the patients who did not receive preoperative bevacizumab treatment, those classified as R1 had a worse OS (*P* = .010). In patients treated with bevacizumab, OS was not significantly associated with margin status (*P* = .081). Since bevacizumab induced a distinct pattern of necrosis and fibrosis, it can be observed on imaging as a lower density of the lesion and the loss of enhancement around the tumor periphery, which suggests that positive margins may have been eliminated.

In conclusion, R1 resection is associated with patients who have more metastases, larger tumors, a greater distribution across different lobes, and a higher proportion of initially unresectable tumors. This indicates a poorer tumor biological behavior and is not an independent predictor of survival. However, due the current prevalence of surgical energetic devices, the use of preoperative neoadjuvant chemotherapy, and postoperative adjuvant chemotherapy and targeted immune therapy, R1 resection is not considered undesirable. For details, please refer to Table 2.

**Table 2:** Prognosis of hepatic R1 margins.

Time	Journal	Number of cases	Conclusion	Cause
2007^[21]^	Br J Surg	176	No statistically significant difference in OS and DFS between R1 < 1 mm and R0 group	CUSA scalpel use adds approximately 5 mm of additional margins
2008^[22]^	Ann Surg	436	No statistically significant difference in 5-yr OS and DFS between R1 < 1 mm and R0 groups	CUSA scalpel dissection of hepatic parenchyma, bipolar coagulation, and argon knife for trabecular hemostasis
2013^[23]^	Arq Bras Cir Dig	91	No statistically significant difference in OS and DFS between margins < 1 mm and ≥ 1 mm groups	Use of surgical energy devices and perioperative chemotherapy
2014^[24]^	Surg Endosc	114	DFS was significantly lower in the R1 < 1 mm group, but there was no difference in OS compared to the R0 group	Repeat hepatic resection after recurrence may have contributed to the overall OS not being different
2015^[27]^	J Gastrointest Surg	332	R1 re-resection to achieve R0 status did not improve long-term prognosis, and R1 was not a risk factor for OS and DFS.	Application of systemic chemotherapy
2011^[28]^	Eur J Surg Oncol	310	No difference in OS and DFS between R1 and R0 in patients who were initially inoperable but received preoperative chemotherapy	Application of preoperative neoadjuvant chemotherapy
2017^[29]^	Br J Surg	630	No difference in OS between R1 and R0 in patients treated with bevacizumab	Application of adjuvant therapy with bevacizumab

CUSA = cavitron ultrasonic surgical aspirator, DFS = disease free survival, OS = overall survival.

## 4. Significance of positive margins in specific cases

The liver has a complex anatomy, including multiple ductal structures, such as the hepatic artery, portal vein, and hepatic vein. Consequently, some tumors may be situated near blood vessels. To preserve these blood vessels, many patients are unable to receive R0 resection. In addition, there are often multiple CRC liver metastases. In cases with multiple foci, excessive resection can leave the liver with insufficient volume. Therefore, an excessive pursuit of R0 resection may hinder the optimal surgical treatment of multiple intrahepatic metastases. Is R0 resection required for lesions located close to blood vessels? Must all multiple lesions undergo R0 resection? Does the prognostic impact of an R1 margin depend on the primary tumor location?

A study by McVey et al concluded that the effect of the margin of excision on OS in patients diagnosed with CRC varies based on the primary site of the cancer.^[30]^ They divided 732 patients diagnosed with CRC who underwent hepatectomy at the Cleveland Clinic and Johns Hopkins Clinic between 2002 to 2016 into 2 groups: those with left-sided colon cancer and those with right-sided colon cancer. After adjusting for other confounding variables, it was determined that a margin of R0 (≥1 mm) was a statistically significant predictor of left-sided colon cancer (HR = 0.629, *P* = .024). However, the R0 margin was not statistically significant for right-sided colon cancer (HR = 0.788, *P* = .245). They discovered that patients with right-sided colon cancer had a higher incidence of KRAS mutations than patients with left-sided colon cancer. Therefore, the biological behavior of the tumor was deemed to be more significant in determining the prognosis of right-sided colon cancer.

In cases of liver metastases, certain lesions are found near crucial blood vessels. If these lesions are removed forcefully, it may result in hemorrhage. Whereas, preserving the blood vessels may not guarantee R0 resection.

Are positive margins considered to be the same for blood vessels in close proximity as they are for the immediate hepatic parenchyma? Viganò et al conducted a prospective study on 627 surgical areas in 226 patients diagnosed with CRLM.^[31]^ They categorized the positive margins into 2 groups: R1 (<1 mm) near blood vessels, and R1 (<1 mm) near the liver parenchyma. It was discovered that the intrahepatic recurrence rate was as high as 49.5% in the group with R1 margins in close proximity to the liver parenchyma, which was significantly higher than the rate of 37.5% in the group with R1 margins adjacent to blood vessels and 35.8% in the group with R0 margins (*P* = .042). The 5-year survival rate in the group with R1 margins in close proximity to the hepatic parenchymal was 32.5%, which was significantly lower than the 59.4% in the group with R1 margins in close proximity to the blood vessels and the 54.3% in the group with R0 margins (*P* = .023). The 5-year survival rates of patients with both vascular R1 margins and hepatic parenchymal R1 margins were similar to those of the hepatic parenchymal R1 margin group (26.7%), and both were significantly lower than those of the R0 margin group (*P* = .026). The hepatic parenchymal R1 margin was identified as an independent risk factor for poor prognosis in the multifactorial regression analysis. However, there was no significant difference in prognosis between the R1 margin group in close proximity to blood vessels and the R0 margin group. Their study confirms the hypothesis that vessels in contact with CRLM limit the spread of tumors. Therefore, when dealing with liver metastases located near major blood vessels, prioritizing the protection of the vessels is more crucial than pursuing an R0 resection.

CRLM typically manifests as multiple metastases, and excessive resection may leave the liver with insufficient volume. Therefore, it is not recommended to pursue R0 resection aggressively. Is it necessary to focus on R1 in all liver lesions when multiple lesions exist? Several studies have demonstrated that liver metastases typically manifest as micrometastases within 4 mm.^[17]^ However, the size of the lesion may have an effect on micrometastasis. Sasaki et al concluded that the likelihood of a lesion developing micrometastases increases with its size.^[32]^ Between 2000 and 2015, they collected clinical data from 251 patients who underwent hepatectomy for multiple liver metastases from CRC. The R1 margins were further subdivided into 2 types: non-maximal R1 lesion type and maximal R1 lesion type. There was no statistically significant difference between the local recurrence rates of 18.8% in the non-maximal lesion type R1 group and 19.2% in the maximal lesion type R1 group. The OS of the non-maximal lesion type R1 group (53.3 months) was comparable to that of the R0 group (66.6 months), whereas the OS of the maximal lesion type R1 group was significantly lower at only 36.5 months (*P* = .037). In the multivariate analysis, only the maximal lesion type R1 was determined to be an independent risk factor for patient prognosis. Therefore, when dealing with multiple lesions, it is essential to prioritize maximal R0 resection of the lesions while also considering the remaining liver volume. Of course, not many studies have been reported in this area, and further studies are warranted for validation.

The relationship between tumor margins and prognosis are also influenced by tumor biological behavior. The study conducted by Margonis et al revealed that the impact of margin cutting on prognosis varied across different genetic conditions.^[33]^ In wtKRAS (wild-type) patients, OS was worse in the R1 (<1 mm) margin group, but there was no significant difference in OS among those with margins of 1 to 4 mm, 5 to 9 mm, and 10 mm or more. In mutKRAS patients (mutant), the 5-year OS was 40.7% in the R1 margin group and 47.9% in the R0 margin group, with no statistically significant difference between them (*P* = .470). Wang et al assessed the tumors based on their genetic and morphological characteristics using the genetic and morphological evaluation score.^[34]^ They discovered that in the low-intermediate-risk group (score 0–3), R1 margins were associated with lower OS and recurrence-free survival rates. However, in the high-risk group (score 4), there was no significant difference in median OS and recurrence-free survival rates between patients who underwent R0 and R1 resection. This suggests that the relationship between the R1 margin and prognosis is tumor-specific.

In conclusion, it is important to prioritize R0 resection whenever possible for patients with left-sided colon cancer. When the CRLM is adjacent to blood vessels and it is necessary to preserve the blood vessels, surgical resection must be considered. If the CRLM is present as multiple metastases, it is crucial to avoid excessive resection that could result in an inadequate liver volume. To achieve an R0 resection, it is essential to ensure that the largest lesion is completely removed.

## 5. Can the positive margins be further reduced

In a survey conducted by Viganò et al regarding the incidence rate, clinical impact, and management of CRLM after hepatectomy, 276 surgeons from 52 countries were surveyed.^[35]^ The survey revealed divided opinions among the surgeons regarding the 1 mm positive margin of excision. Only half of the surgeons believed that defining the R1 excision as < 1 mm was acceptable, while the other half believed that a 0 to 1 mm margin could be considered acceptable to define R1 as 0 mm under the microscope. In recent years, the results of studies with R1 of 0 mm have been reported. Ayez et al determined R1 as 0 mm based on a stratified analysis of 264 patients admitted between January 2000 and December 2008.^[36]^ They divided the patients into 2 subgroups: those who received neoadjuvant chemotherapy and those who did not. Patients who did not receive neoadjuvant chemotherapy had a significantly longer median DFS in the R0 group (17 months) than in the R1 group (8 months) (*P* < .001). In addition, the median OS of the 2 groups, the R0 group (53 months) and the R1 group (30 months), was statistically significant (*P* < .001). In patients who received neoadjuvant chemotherapy, the difference in median DFS (*P* = .303) and median OS (*P* = .645) between R0 and R1 resections was not statistically significant, and neoadjuvant chemotherapy eliminated the difference between the R1 and R0 groups. In the study conducted by Mbah et al, patients were divided into 4 subgroups for comparison based on parenchymal margins^[37]^: positive R1 (0 mm) and negative R0 margins. The negative margins were further divided into groups with margins < 1 mm, 1 to 10 mm, and > 10 mm. The R1 margin group had the shortest median survival and DFS, while there was no significant difference between the negative margin groups in terms of OS and DFS. Ausania et al^[38]^ investigated the effect of R1 margins on postoperative recurrence, specifically comparing R1 margins of 0 with margins <1 mm. They concluded that the median DFS was significantly higher in the group with R1 margins <1 mm compared to the group with R1 margins of 0 (93 vs 55 months, *P* = .025). In addition, disease progression-free survival was significantly longer in the group with R1 margins of <1 mm compared to the group with R1 margins of 0 (69 vs 46 months, *P* = .038). In addition, they discovered that R1 margins of 0 were the only independent risk factor associated with higher margin recurrence (OR = 5.6, *P* = .046). Laurent et al defined an R1 margin of 0 mm in a study of 466 patients who underwent hepatectomy for CRLM.^[39]^ They revealed that in the modern era of effective chemotherapy, R1 resection did not affect OS and DFS in patients who responded to neoadjuvant chemotherapy, despite contributing to an increase in intrahepatic recurrences. Moreover, regardless of the status of marginal resection, effective postoperative chemotherapy can aid in the prevention of recurrence. Clearly, several studies have narrowed the boundaries of R1 even further.

## 6. Conclusion

Regarding the margins of liver resection in CRLM, there are still many controversies, and the overall trend is gradually shifting from the original “1 cm guideline” to the current > 1 mm R0 resection. However, most scholars continue to reject R1 resection.^[40]^ R1 resection is believed to have a worse prognosis than R0 resection, and the DFS after surgery is shorter.^[41–44]^ Since CRLM often occurs in multiple locations and has a high likelihood of recurrence after surgery, it is essential to preserve as much healthy liver tissue as possible while effectively removing the tumor. This is essential for future re-resection in case of recurrence and for preventing postoperative liver failure. Consequently, parenchyma-preserving hepatectomy is preferrable.^[45]^ Determining the margins of incision to preserve as much liver parenchyma as possible without compromising the prognosis is a crucial aspect of this procedure. Although there have been numerous studies and reports, patients who undergo R1 resection typically have larger tumors, more metastatic lesions, and a wider distribution. Consequently, the surgery is more complex, and achieving the desired homogeneity is more difficult than with R0 resection. Therefore, further in-depth research is required. Additionally, laparoscopic surgery has grown in popularity. However, achieving R0 resection laparoscopically when dealing with larger tumors or tumors located in specific areas can be challenging. Therefore, additional research on liver margins can provide a more solid basis for laparoscopic surgery.

## Author contributions

**Conceptualization:** Xiang-Nan Ai, Qiang Zhang, Dian-Rong Xiu.

**Data curation:** Xiang-Nan Ai, Chang-Guo Jin, Hao Hu, Wen-Xuan Zhang, Zhen-Yu Wu.

**Formal analysis:** Hao Hu, Wen-Xuan Zhang, Zhen-Yu Wu.

**Writing – original draft:** Xiang-Nan Ai, Qiang Zhang, Chang-Guo Jin, Dian-Rong Xiu.
